# Risk Factors of End Stage Renal Disease in Peshawar, Pakistan: Odds Ratio Analysis

**DOI:** 10.3889/oamjms.2016.068

**Published:** 2016-07-10

**Authors:** Salahuddin Khan, Tariq Hussain, Najma Salahuddin, Salahuddin Mehreen

**Affiliations:** 1 *Department of Family and Community Medicine, College of Medicine, University of Dammam, Kingdom of Saudi Arabia*; 2 *Higher Education Department, Peshawar, Pakistan*; 3 *Department of Statistics, Shaheed Benazir Bhutto Women University, Peshawar, Pakistan*; 4 *Medical Officer, North West Hospital, Phase 5 Hayatabad, Peshawar, Pakistan*

**Keywords:** Odds ratio, confidence interval, ESRD, risk factors, association

## Abstract

**AIM:**

The basic aim of this study was to discover the association of End Stage Renal Disease (ESRD) with various risk factors. End Stage Renal Failure is the last stage of the chronic renal failure in which kidneys become completely fail to function.

**MATERIALS AND METHODS:**

The data were collected from the patients of renal diseases from three major hospitals in Peshawar, Pakistan. Odds ratio analysis was performed to examine the relationship of ESRD (a binary response variable) with various risk factors: Gender, Diabetic, Hypertension, Glomerulonephritis, Obstructive Nephropathy, Polycystic kidney disease, Myeloma, SLE Nephritis, Heredity, Hepatitis, Excess use of Drugs, heart problem and Anemia.

**RESULTS:**

Using odds ratio analysis, the authors found that the ESRD in diabetic patients was 11.04 times more than non-diabetic patients and the ESRD were 7.29 times less in non-hypertensive patients as compared to hypertensive patients. Similarly, glomerulonephritis patients had 3.115 times more risk of having ESRD than non-glomerulonephritis. Other risk factors may also, to some extent, were causes of ESRD but turned out insignificant due to stochastic sample.

**CONCLUSION:**

The authors concluded that there is a strong association between ESRD and three risk factors, namely diabetes, hypertension and glomerulonephritis.

## Introduction

Statistical methods are applied frequently in medical research, which deals with issues that are of great concern for the general public. It is now a well-known fact that no research could be carried out without having sufficient knowledge of Statistics. Particularly, medical research requires a good understanding of statistical methods.

End Stage Renal Disease (ESRD) is the last stage of the chronic renal failure in which kidneys fail to function completely. At this stage, the kidney stops its functions to remove the impurities and control electrolytes. The symptoms of ESRD comprise less urine output, swelling of legs, face, nausea and vomiting [1].

ESRD is one of the major health problems throughout the world. Several investigations have been carried out to study various risk factors of the ESRD. The United States Renal Data System (USRDS) established in 1989. This system is the largest and most comprehensive national Chronic Kidney Disease (CKD) and ESRD surveillance system [2]. The death rate due to ESRD in western countries especially in the USA is higher, but in Asian countries like Pakistan, India, Bangladesh; there are also a significant number of deaths due to ESRD [3].

It has been established that both low estimated Glomerular Filtration Rate (eGFR) and high albuminuria were independently associated with mortality and ESRD regardless of age across a wide range of populations [4]. A retrospective cross-sectional study was conducted to investigate the prevalence and associated comorbidities of Stage 3 (GFR 30-59 ml/min/1.73 m^2) and Stages 4 and 5 (GFR <30 mL/min/1.73 m^2) CKD among Chinese nursing home older adults. The researchers concluded that stages 3 to 5 CKD are widespread in Chinese nursing home older adults [5]. Regardless of higher risks of mortality and ESRD in diabetes, the relative risks of these outcomes by eGFR and Albumin-to-Creatinine Ratio (ACR) are much the same irrespective of the presence or absence of diabetes, highlighting the significance of kidney disease as a predictor of clinical outcomes [6].

It has been recognized that males and females face increased risk of all-cause mortality, cardiovascular mortality, and ESRD with lower estimated GFR and higher albuminuria [7]. It has been shown that declines in estimated GFR smaller than a doubling of serum creatinine concentration occurred more commonly and were strongly and consistently associated with the risk of ESRD and mortality, supporting consideration of lesser declines in estimated GFR (such as a 30% reduction over 2 years) as an alternative end point for CKD development [8]. A kidney failure definition, including treated and untreated disease identifies more cases than linkage to the United States Renal Data System registry alone, particularly among older adults [9]. It has been found that CKD is increasingly common in older adults. Competing risks of death influence the risk of development to ESRD [10].

By using survival analysis through the Cox proportional hazard model, the researchers found that the elevated C - reactive protein (CRP) was a robust predictor of mortality in ESRD patients. In a study of 663 ESRD patients (374 males and 289 females), the researchers also found that CRP was a strong predictor. CRP had positive correlation (= 0.369; p-value equal to 0.001) in addition, the coefficient of correlation for females (= 0.519; p-value < 0.0001) and male correlation (= 0.372; p-value < 0.0001) [11].

A univariate Cox regression analysis was carried out and the researchers found that the chlamydia pneumonia infection was related to the cardiovascular risk of ESRD patients. In a cohort of 227 ESRD patients, the Hazard Ratio of mortality was 1.08 with 95% confidence interval (0.678 to 1.722); p-value = 0.737. The researchers concluded that the chlamydia pneumonia infection is a major risk factor in patients with ESRD [12].

In a prospective cohort study of 143802 patients in China, having an age of 40 years or above, the researchers found that the Body Mass Index (BMI) is strongly associated with ESRD. The multivariate-adjusted risks for ESRD are 1.389 with 95% confidence interval (1.021 to 1.909) for BMI < 18.52 kg/m, 1.213 with 95% confidence interval (0.919 to 1.598) for BMI (25.01 to 29.93 kg/m) and 2.142 with confidence interval 95% (1.392 to 3.289) for BMI ≥ 30.01 kg/m with J-shaped association [13]. A cohort study was carried out in survival analysis and the researchers concluded that the independent risk factors of ESRD are sex, race, anemia and heredity [14].

In this study, odds ratio analysis was used to examine the relationship of ESRD with various risk factors.

## Materials and Methods

To determine the effects of various risk factors on ESRD, this study was carried out based on the data obtained from three major hospitals in Peshawar: (i) Hayatabad Medical Complex, (ii) Lady Reading Hospital and (iii) Khyber Teaching Hospital. The association of various risk factors with the occurrence of ESRD was determined through the statistical technique of odds ratio analysis. A total of 407 patients was examined for the presence or absence of ESRD. The statistical analyses were performed with SPSS software package.

**Odds and the odds ratio:** The probability of interested events divided by the probability of non-interested events are called the Odds, i.e. Odd = P/1-P, where P is the probability of interested events. If the observed dichotomous data contain ‘X’ number of interested events in ‘n’ outcomes, then the odds ratio of interest can be calculated as:


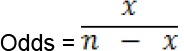


‘X’ denotes the number of occurrences of interested events and ‘n-X’ indicates the number of non-interested events.

In order to compare two binary data sets, the ratio of odds of interest in one set to the odds of the other data set, is a relative measure of odds of interest. The odds ratio is denoted by, and mathematically, it is defined as:


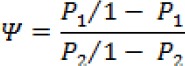


If the probability of interest in two data sets is equal, then the odds ratio (Ψ) = 1 and if odd ratio (Ψ) < 1, then the odds of interest will be less in the first data set than in the second one. On the other hand, if the odds ratio (Ψ) > 1, then the odds of an interest will be greater in the first data set [15].

**Statistical inference based on odds ratio:** To estimate the odds ratio, the binary data are needed to arrange in (2x2) contingency table given as:

**Contingency Table**

**Table:** 

	No. of Success	No. of Failure	Total
Data Set 1	A	B	a+b
Data Set 2	C	D	c+d
Total	a+c	b+d	N

The probabilities of interest obtained from two data sets are 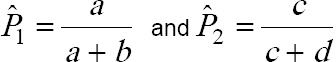 and 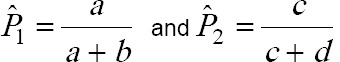. The estimated odds ratio (ψ) is given by:


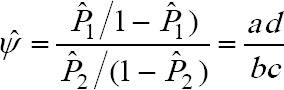


This estimated odds ratio (ψ̂) is usually termed as “cross-product ratio”, as it is obtained by multiplying the two pairs of diagonal values in the (2 x 2) contingency table [16].

To test such an association, the hypothesis is considered as:

H_0_:*ψ* = 1 or equivalently, H_0_: 1n(*ψ*) = 0, means that the two variables (ESRD and Risk factors) are independent, that is, risk factors do not affect ESRD.

The test-statistic is: 
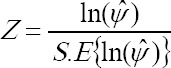
, which has an approximate standard normal distribution. An approximate 100 (1-α) % confidence interval for 1n(*ψ*) is constructed as:


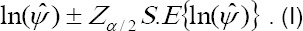


For example, a 95% confidence interval for 1n(*ψ*) is given by


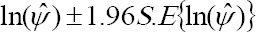


The confidence interval given by equation (I) on inversion will give us the confidence interval for *ψ* as *ψ̂e*^-*z*α/2*S.E*^ < ψ < *ψ̂*^*z*α/2*S.E*^:

If the interval contains unity, it indicates independence; otherwise an association between risk factor and ESRD is significant.

## Results

Several researchers have investigated the association of ESRD and its various risk factors. A meta-analysis study was conducted. The interpretation of this study was that CKD should be regarded as at least an equally relevant risk factor for mortality. These researchers further interpreted that ESRD in individuals without hypertension should be regarded as it is in those with hypertension [17]. It has been revealed that diabetes, higher systolic blood pressure, lower estimated glomerular filtration rate and black race were risk factors for developing treated chronic kidney failure irrespective of albuminuria status, although the absolute risk of kidney failure in participants without albuminuria was very low. These researcher also showed that their findings support testing for kidney disease in high-risk populations, which often have otherwise unrecognized kidney disease [18].

To investigate the relationship of ESRD with various risk factors, we used odds ratio analysis.

### ESRD versus gender

Contingency table of ESRD versus gender is given in Table (1). The calculated values are: Odd Ratio = 1.40, Chi-square = 2.628, p-value = 0.105 and the confidence interval is (0.931, 2.104).

**Table 1:** Contingency table of ESRD versus gender

Gender	ESRD		Total (%)
	No	Yes	
*Female*	104	59	163 (40)
Male	136	108	244 (60)
Total (%)	240 (59)	167 (41)	407

The odds of ESRD show that the males are 1.4 times more exposed to ESRD than the females and the Log of the odds ratio is 0.336 (with a standard error = 0.208). The confidence interval for the odds ratio is (0.931, 2.104) at the 5% level of significance. The interval contains unity; it indicates independence (no association between Gender and ESRD). Also, p-value is greater than 0.05, the result is insignificant. It is concluded that there is no association between Gender and ESRD.

### ESRD versus diabetic

Contingency table of ESRD versus diabetic is given in Table 2. The calculated values are: Odd Ratio = 11.04, Chi-square = 141.883, p-value < 0.001 and the confidence interval is (6.913, 17.63).

**Table 2:** Contingency table of ESRD versus diabetic

Diabetic	ESRD		Total (%)
	No	Yes	
*No*	185	39	224 (55)
Yes	55	128	183 (45)
Total (%)	240 (59)	167 (41)	407

The odds of ESRD show that the diabetic patients are 11.04 times more exposed to ESRD than the non-diabetic patients and the Log of odds ratio is 2.402 (with a standard error = 0.239). The confidence interval for the odds ratio is (6.913, 17.63) at the 5% level of significance. The interval does not contain unity; it indicates that there is an association between Diabetic and ESRD. Also, observed p-value is less than 0.05, the result is significant. It is concluded that there is a strong association between diabetes and ESRD.

### ESRD versus hypertension

Contingency table of ESRD versus hypertension is given in Table 3. The calculated values are: Odd Ratio = 7.287, Chi-square = 77.56, p-value < 0.001 and the confidence interval is (4.571, 11.616).

**Table 3:** Contingency table of ESRD versus hypertension

Hypertension	ESRD		Total (%)
	No	Yes	
*No*	152	32	184 (45)
Yes	88	135	233 (55)
Total (%)	240 (59)	167 (41)	407

The odds of ESRD show that the hypertensive patients are 7.287 times more exposed to ESRD than the non-hypertensive patients and the Log of odds ratio is 1.986 (with a standard error = 0.238). The confidence interval for the odds ratio is (4.571, 11.616) at the 5% level of significance. The interval does not contain unity; it indicates there is an association between hypertension and ESRD. Also, observed p-value is less than 0.05, the result is significant. It is concluded that there is a strong association between hypertension and ESRD.

### ESRD versus glomerulonephritis

Contingency table of ESRD versus glomerulonephritis is given in Table 4. The calculated values are: Odd Ratio = 3.115, Chi-square = 29.826, p-value < 0.001 and the confidence interval is (2.059, 4.712).

**Table 4:** Contingency table of ESRD versus glomerulonephritis

Glomerulonephritis	ESRD		Total (%)
	No	Yes	
No	171	74	245 (60)
Yes	69	93	162 (40)
Total (%)	240 (59)	167 (41)	407

The odds of ESRD show that the glomerulonephritis patients are 3.115 times more exposed to ESRD than the non-glomerulonephritis patients and the Log of odds ratio is 1.136 (with a standard error = 0.211). The confidence interval for the odds ratio is (2.059, 4.712) at the 5% level of significance. The interval does not contain unity; it indicates there is association between glomerulonephritis and ESRD. Also, observed p-value is less than 0.05, the result is significant. It is concluded that there is a strong association between glomerulonephritis and ESRD.

### ESRD versus obstructive nephropathy

Contingency table of ESRD versus obstructive nephropathy is given in Table 5. The calculated values are: Odd Ratio = 1.2, Chi-square = 0.542, p-value = 0.462 and the confidence interval is (0.738, 1.952).

**Table 5:** Contingency table of ESRD versus obstructive nephropathy

Obstructive Nephropathy	ESRD		Total (%)
	No	Yes	
*No*	194	130	324 (80)
Yes	46	37	83 (20)
Total (%)	240 (59)	167 (41)	407

The odds of ESRD show that the obstructive nephropathy patients are 1.2 times more exposed to ESRD than the non-obstructive nephropathy patients and the Log of odds ratio is 0.182 (with standard error = 0.248). The confidence interval for the odds ratio is (0.738, 1.952) at the 5% level of significance. The interval contains unity; it indicates independence. Also, observed p-value is greater than 0.05, the result is insignificant. It is concluded that there is no association between obstructive nephropathy and ESRD.

### ESRD versus polycystic kidney disease

Contingency table of ESRD versus Polycystic kidney is given in Table 6. The calculated values are: Odd Ratio = 1.67, Chi-square = 0.186, p-value = 0.403 and Confidence Interval is (0.553, 2.527).

**Table 6:** Contingency table of ESRD versus polycystic kidney disease

APKD	ESRD		Total (%)
	No	Yes	
*No*	224	154	378 (93)
Yes	16	13	29 (7)
Total (%)	240 (59)	167 (41)	407

The odds of ESRD show that the patients, who had Polycystic kidney disease, are 1.67 times more exposed to ESRD than the patients who do not have Polycystic kidney disease and the Log of odds ratio is 0.167 (with standard error = 0.288). The confidence interval for the odds ratio is (0.553, 2.527) at the 5% level of significance. The interval contains unity; it indicates independence. Also, observed p-value is greater than 0.05, the result is insignificant. It is concluded that there is no association between polycystic kidneydisease and ESRD.

### ESRD versus myeloma

Contingency table of ESRD versus myeloma is given in Table 7. The calculated values are: Odd Ratio = 1.081, Chi-square = 0.20, p-value = 1.000 and Confidence Interval is (0.368, 3.174).

**Table 7:** Contingency table of ESRD verses myeloma

Myeloma	ESRD		Total (%)
	No	Yes	
*No*	232	161	393 (97)
Yes	8	6	14 (3)
Total (%)	240 (59)	167 (41)	407

The odds of ESRD show that the myeloma patients are 1.081 times more exposed to ESRD than the non-myeloma patients and the Log of odds ratio is 0.078 (with a standard error = 0.302). The confidence interval for the odds ratio is (0.368, 3.174) at the 5% level of significance. The interval contains unity; it indicates independence. Also, observed p-value is greater than 0.05, the result is insignificant. It is concluded that there is no association between myeloma and ESRD.

### ESRD versus SLE nephritis

Contingency table of ESRD versus SLE nephritis is given in Table 8. The calculated values are: Odd Ratio = 1.132, Chi-square = 0.051, p-value = 0.802 and Confidence Interval is (0.41, 3.077).

**Table 8:** Contingency table of ESRD versus SLE nephritis

SLE Nephritis	ESRD		Total (%)
	No	Yes	
*No*	231	160	391 (96)
Yes	9	7	16 (4)
Total (%)	240 (59)	167 (41)	407

The odds of ESRD show that the SLE nephritis patients are 1.123 times more exposed to ESRD than the non-SLE nephritis patients and the Log of odds ratio is 0.116 (with standard error = 0.551). The confidence interval for the odds ratio is (0.41, 3.077) at the 5% level of significance. The interval contains unity; it indicates independence. Also, observed p-value is greater than 0.05, the result is insignificant. It is concluded that there is no association between SLE nephritis and ESRD.

### ESRD versus heredity

Contingency table of ESRD versus heredity is given in Table 9. The calculated values are: Odd Ratio = 1.757, Chi-square = 1.818, p-value = 0.202 and Confidence Interval is (0.767, 4.024).

**Table 9:** Contingency table of ESRD versus heredity

Heredity	ESRD		Total (%)
	No	Yes	
*No*	229	154	383 (94)
Yes	11	13	24 (6)
Total (%)	240 (59)	167 (41)	407

The odds of ESRD show that the patients, who have a family history of ESRD, are 1.757 times more exposed to ESRD than the patients who do not have a family history of ESRD and the Log of odds ratio is 0.564 (with standard error = 0.423). The confidence interval for the odds ratio is (0.767, 4.024) at the 5% level of significance. The interval contains unity; it indicates independence. Also, p-value is greater than 0.05, the result is insignificant. It is concluded that there is no association between heredity and ESRD.

### ESRD versus hepatitis

Contingency table of ESRD versus hepatitis is given in Table 10. The calculated values are: Odd Ratio = 1.792, Chi-square = 4.495, p-value = 0.063 and Confidence Interval is (0.747, 2.277).

**Table 10:** Contingency table of ESRD versus hepatitis

Hepatitis	ESRD		Total (%)
	No	Yes	
*No*	211	134	345 (85)
Yes	29	33	62 (15)
Total (%)	240 (59)	167 (41)	407

The odds of ESRD show that the hepatitis patients are 1.792 times more exposed to ESRD than the non-hepatitis patients and the Log of odds ratio is 0.253 (with a standard error = 0.277). The confidence interval for the odds ratio is (0.747, 2.277) at the 5 % level of significance. The interval contains unity; it indicates independence. Also, observed p-value is greater than 0.05, the result is insignificant. It is concluded that there is no association between hepatitis and ESRD.

### ESRD versus drug usage

Contingency table of ESRD versus drug usage is given in Table 11. The calculated values are: Odd Ratio = 1.157, Chi-square = 0.091, p-value = 0.809 and Confidence Interval is (0.100, 2.994).

**Table 11:** Contingency table of ESRD versus drug usage

Hepatitis	ESRD		Total (%)
	No	Yes	
*No*	230	159	389 (96)
Yes	10	8	18 (4)
Total (%)	240 (59)	167 (41)	407

The odds of ESRD show that the patients, who used a lot of drugs, are 1.157 times more exposed to ESRD than the patients who do not use a lot of drugs and the Log of odds ratio is 0.146 (with standard error = 0.485). The confidence interval for the odds ratio is (0.100, 2.994) at the 5% level of significance. The interval contains unity; it indicates independence. Also, observed p-value is greater than 0.05, the result is insignificant. It is concluded that there is no association between drug usage and ESRD.

### ESRD versus heart problem

Contingency table of ESRD versus heart problem is given in Table 12. The calculated values are: Odd Ratio = 1.15, Chi-square = 0.0243, p-value = 0.672 and Confidence Interval is (0.100, 2.994).

**Table 12:** Contingency table of ESRD verses heart problem

Heart Problem	ESRD		Total (%)
	No	Yes	
*No*	227	156	383 (94)
Yes	13	11	24 (6)
Total (%)	240 (59)	167 (41)	407

The odds of ESRD show that the patients who have heart problem are 1.231 times more exposed to ESRD than the patients who do not have heart problems and the Log of the odds ratio is 0.208 (with standard error = 0.422). The confidence interval for the odds ratio is (0.543, 2.285) at the 5% level of significance. The interval contains unity; it indicates independence. Also, observed p-value is greater than 0.05, the result is insignificant. It is concluded that there is no association between heart problem and ESRD.

### ESRD versus anemia

Contingency table of ESRD versus anemia is given in Table 13. The calculated values are: Odd Ratio = 1.083, Chi-square = 0.088, p-value = 0.788 and Confidence Interval is (0.676, 4.024).

**Table 13:** Contingency table of ESRD verses anemia

Anemia	ESRD		Total (%)
	No	Yes	
*No*	201	138	339 (83)
Yes	39	29	68 (17)
Total (%)	240 (59)	167 (41)	407

The odds of ESRD show that the anemia, patients are 1.083 times more exposed to ESRD than the non-anemia patients and the Log of odds ratio is 0.080 (with a standard error = 0.269). The confidence interval for the odds ratio is (0.767, 4.024) at the 5 % level of significance.

The interval contains unity; it indicates independence. Also, observed p-value is greater than 0.05, the result is insignificant. It is concluded that there is no association between anemia and ESRD.

## Discussion

The major aim of this study was to determine the most important risk factors of ESRD in Peshawar. A total of 407 patients was examined in the three major hospitals of Peshawar and the phenomena of ESRD was studied in relation to different risk factors like diabetic, hypertension, glomerulonephritis, obstructive nephropathy, polycystic kidney diseases, myeloma, SLE nephritis, heredity, hepatitis, excess use of drugs, heart problem and anemia.

Out of 407 patients, 244 (60%) were males and 163 (40%) were females. The average age of male patients was 43.38 years and the average age of female patients was 42.4 years. Out of 244 male patients, 108 patients were in an uncontrolled group (ESRD cases) and out of 163 female patients, 59 were in an uncontrolled group (ESRD cases).

The total number of diabetic patients was 183 in which 128 patients had ESRD. On the other hand, out of 185 non-diabetic patients, 39 patients had ESRD. The total number of 233 (55%) patients had hypertension in which 135 (60.5%) had ESRD and out of 184 non-hypertension patients, 32 patients had ESRD. A total of 162 patients had glomerulonephritis in which 93 (57.4%), patients had ESRD and 74 patients out of 240 non-glomerulonephritis patients, had ESRD. The total number of 62 patients had hepatitis, in which 33 patients had ESRD and 134 patients out of 345 non-hepatitis patients, had ESRD.

The total number of 83 patients had obstructive nephropathy problem in which 37 patients had ESRD. On the other hand, 120 patients out of 324 non-obstructive nephropathy patients had ESRD. The total number of 29 patients had polycystic kidney disease problem in which 13 have ESRD. On the other hand, 154 patients out of 324 non-polycystic kidney disease patients had ESRD. There were 14 myeloma patients out of 407, in which 6 patients had ESRD. On the other hand, 161 patients out of 378 non-myeloma patients had ESRD. There were 16 SLE nephropathy patients out of 407, in which 7 had ESRD. On the other hand, 160 patients out of 393 non-SLE nephropathy patients had ESRD. A total of 13 patients out of 24 patients had a family history of ESRD. On the other hand, 154 patients out of 389 non-heredity patients had ESRD. There were 18 patients who used a lot of drugs in which 8 patients had ESRD. On the other hand, 159 patients out of 384 non-drug user had ESRD. A total of 24 patients had heart problems in which 11 had ESRD. On the other hand, out of 383 non-heart problem patients, 156 patients had ESRD. Out of 68 anemic patients, 29 patients had ESRD. On the other hand, 138 patients out of 339 non-anemia patients had ESRD.

Using odds ratio analysis, it was found that the ESRD in diabetic patients were 11.04 times more than non-diabetic patients and the ESRD in hypertensive patients were 7.29 times more than non-hypertensive patients. Similarly, glomerulonephritis patients have 3.115 times more chances to have ESRD than non-glomerulonephritis. This analysis shows that there was a strong association between ESRD and the three risk factors diabetes, hypertension and glomerulonephritis. The odds of ESRD for heredity were 1.76 times more than non-heredity patients. The odds of ESRD for non-hepatitis patients were 1.792 times less than hepatitis patients.

Based on odds ratio analysis, using data from 407 patients from three major hospitals of Peshawar, the researchers concluded that the main causes of ESRD were the three risk factors i.e. diabetes, hypertension and glomerulonephritis. Other risk factors, i.e. obstructive nephropathy, heredity and hepatitis may also, to some extent, causes of ESRD but in this study, it turned out to be insignificant due to stochastic sample.

The researchers concluded that the main finding of this study is that there is a strong association between ESRD and the three risk factors namely diabetic, hypertension & glomerulonephritis.
